# Community engagement in public health palliative care: A comparative ethnographic study of two culturally distinct compassionate communities in Canada

**DOI:** 10.1177/26323524251400806

**Published:** 2025-12-15

**Authors:** Émilie Lessard, Isabelle Marcoux, Serge Daneault, Lise Jean, Cynthia Lapointe, Dale Weil, Ghislaine Rouly, Libby Sallnow, Allan Kellehear, Antoine Boivin

**Affiliations:** 1 Institute of Psychology, Faculty of Social and Political Sciences, Université de Lausanne, Switzerland; 2 Faculty of Health Science, University of Ottawa, ON, Canada; 3 Department of Family Medicine and Emergency Medicine, Faculty of Medicine, Université de Montréal, QC, Canada; 4 Centre de recherche de l’Institut universitaire de gériatrie de Montréal (CRIUGM), QC, Canada; 5 Communauté Compatissante Montréal, QC, Canada; 6 Montreal Institute for Palliative Care, Teresa Dellar Palliative Care Residence, Kirkland, QC, Canada; 7 Canada Research Chair in Partnership with Patients and Communities, Centre de recherche du centre hospitalier de l’Université de Montréal (CRCHUM), QC, Canada; 8 Marie Curie Palliative Care Research Department, University College London, UK; 9 Social Work, Education and Community Wellbeing, Northumbria University, Newcastle, UK

**Keywords:** community engagement, comparative study, compassionate communities, ethnography, evaluation, public health palliative care

## Abstract

**Background:**

Compassionate communities are part of an international public health movement aiming to address social determinants of death by fostering supportive environments. Yet, empirical research on community engagement in this field is still limited, particularly the influence of local contexts on engagement patterns.

**Objectives:**

This study aimed to identify effective engagement practices and contextual factors influencing the development and sustainability of compassionate communities.

**Research design and method:**

A comparative ethnographic method was used to examine community engagement processes in two culturally distinct compassionate communities in Montréal (Canada): Centre-Sud and West Island. Data collection included participant observation, semistructured interviews, and logbooks. Informed by developmental evaluation, the analysis was guided by a thematic lens approach and the Ecology of Engagement framework.

**Results:**

Two distinct, context-sensitive paths to community engagement emerged, shaped by the sociocultural realities of each setting. In Centre-Sud, a grassroots, community-led approach focused on shared leadership and building trust fostered a resilient network that achieved sustainability through the creation of an independent nonprofit organization. In contrast, West Island’s institutionally led strategy was a pragmatic response to navigate contextual barriers like preexisting community distrust, achieving sustainability by embedding the initiative within the lead organization via a permanent staff role.

**Conclusion:**

This comparative ethnography demonstrates that success is not defined by a single model but by adapting engagement strategies to local dynamics of trust and power. It highlights that while community-led approaches can foster deep ownership, institutionally led strategies can provide a crucial pathway to sustainability in contexts facing systemic barriers. The study offers a practical framework for practitioners and key lessons for developing evidence-based policy to support compassionate communities in diverse settings.

## Plain language summary

Developing compassionate communities: Lessons from two different Montréal neighborhoods

Developing ‘compassionate communities’—local networks that support people through serious illness, grief, and the end of life—is a shared effort. This research explored the most effective ways to engage communities in this work by comparing two distinct Montréal neighborhoods: the downtown Centre-Sud area and the suburban West Island.

We found that there’s no single recipe for success. In the downtown Centre-Sud area, community groups took the lead, building a strong, trusted network that eventually became its own non-profit organization to ensure sustainability. In the suburban West Island, a local palliative care residence guided the process, creating a permanent staff role to secure a lasting impact.

The key lesson is that the best approach depends on a community’s unique character and history. Success comes from adapting the strategy to fit the local reality.

## Community engagement in compassionate communities

Compassionate communities are part of an international movement aiming to mobilize citizens, community organizations, and civic institutions to address the social and structural determinants of death and dying by creating more supportive environments.^1^ This international movement has grown over three decades, from foundational community-based models in places like Kerala, India, to widespread initiatives now documented in at least 19 countries, including Australia, Canada, the United Kingdom, and the United States.^2^ Despite this global momentum and the fundamental role of community engagement in this model, empirical research remains underdeveloped, hindering evidence-based guidance for practitioners.^3–6^ Consequently, a critical gap persists in understanding how local contexts shape community engagement processes and outcomes within compassionate communities initiatives.

To address this gap, this comparative ethnographic study explores the interplay of local context and engagement processes in two culturally distinct compassionate communities in Montréal, Canada. This sharp sociocultural and linguistic contrast within a single city makes Montréal an ideal ‘natural laboratory’ to examine how engagement strategies must adapt to different community ecosystems. By comparatively analyzing the processes in these two settings, we address the research question: ‘*How does community engagement evolve and influence the development, outcomes, and sustainability of compassionate community initiatives within diverse contexts?*’ This approach, informed by our previous protocol,^6^ allows us to generate transferable insights for other public health palliative care initiatives globally. The resulting programs, perceived social changes and observed transformations from this work are detailed in a separate article.

## Methods

### Research approach and study design

A detailed description of the study design and methodology can be found in our previously published research protocol.^6^ In this study, we used a comparative ethnographic design, which involves the in-depth, immersive study of people within their natural settings to understand their experiences, behaviors, and social interactions. This design was chosen to describe, understand and compare the evolution of community engagement over time, and how the development of compassionate communities varied across culturally diverse settings. Inspired by developmental evaluation,^7^ we documented the community engagement process and development trajectories prospectively in real-time through ethnographic methodology. The reporting of this study conforms to the Standards for Reporting Qualitative Research^8^ statement (see Supplemental File 1).

### Research setting and case selection

The research project, funded by a philanthropic foundation, was co-initiated by the Montréal Institute for Palliative Care and the Canada Research Chair in Partnership with Patients and Communities (CRCHUM). The interdisciplinary action-research team included clinicians, community members, a patient partner, and researchers from diverse disciplines (anthropology, community psychology, management, medicine, public health, sociology), and with complementary content expertise (community engagement, end-of-life care, participatory research, social innovation). To ensure the findings had broader applicability, two contrasting Montréal neighborhoods, *Centre-Sud* and *West Island*, were purposefully selected to clearly delimitate case boundaries, because they have contrasting sociocultural profiles (i.e., English/French cultures, richer/poorer and older/younger than average), making them well-suited for comparative ethnographic research. West Island is the territory attached to the Montréal Institute for Palliative Care. Centre-Sud was chosen for its diverse population and potential to reach marginalized groups, aligning with the research goals.

## Participants

This study involved three primary categories of participants in both sites:

1. *Compassionate community facilitators*: These individuals facilitated community outreach, organizing, co-design of activities, and co-governance support. They played a central role in mobilizing and engaging community members;
2. *Community organization representatives*: This category includes individuals affiliated with various community organizations involved in the development of compassionate community initiatives. For instance, participants at the Centre-Sud Compassionate Community governance table or members of the Community Seniors Table in the West Island;
3. *Citizens, patients, and community members*: This broad category encompasses individuals who participated in various activities related to the compassionate community initiatives, such as citizen forums and other community events.

Eligibility for participation in this study was restricted to adults (18 years of age and older) who were able to provide informed consent. Between February 2021 and December 2023, observations were conducted on over 300 individuals participating in activities related to the development of compassionate community initiatives. Observations included public events (e.g., International Overdose Awareness Day, Citizen Forums) and project-specific activities (e.g., community development meetings, resource coordination tables, compassionate programs). Observations consisted of detailed field notes recorded during each event and activity. Due to the dynamic nature of community engagement, with individuals joining and leaving at various stages, precisely calculating the number of unique participants was not possible in this study.

### Action-research team

This research project was built on a strong collaboration between academic researchers and community partners. An executive committee was established in 2020 to guide the project, comprising academic researchers (A.B., E.L., I.M.), community engagement facilitators (C.L., L.J., S.D.), palliative care leader and project initiator (D.W.), and a patient partner with lived experience of end-of-life care (G.R.). This structure created a space for shared dialogue and decision-making, fostering the integration of everyone’s perspectives and expertise. To facilitate this, 37 meetings were held, totaling 74 h.

To trace the dynamic evolution and varied influences of community engagement, we adopted an emic-etic research approach. Drawing on ethnographic debates regarding researcher positionality—specifically ‘emic’ (insider) and ‘etic’ (outsider) perspectives,^9,10^ individual team members assumed specific roles within this framework, acting as ‘insider’, ‘outsider’, or ‘bridge’ researchers^11^:

- *‘Inside’ researchers (C.L., L.J., S.D., D.W.)*: They facilitated and led community engagement activities, participating in the study both as action-researchers and participants (emic).
- *‘Outside’ researchers (A.B., A.K., G.R., I.M., L.S.)*: As executive committee members with scientific responsibility for the project, they provided research-based guidance (design, methodology, publications, knowledge). They did not participate in community engagement activities, data collection, or analysis (etic).
- *‘Bridge’ researcher (E.L.)*: Acting both as an observer of community engagement processes and participant in the development activities, the lead author collected and analyzed the data as the project unfolded, bridging emic and etic viewpoints by acting as a liaison between the ‘inside’ and ‘outside’ researchers.

This allowed us to capture both lived experiences and external observations, revealing nuanced contextual factors influencing the engagement, development, and sustainability of compassionate communities. This approach mitigates the inherent biases of a purely emic or etic study, fostering a more holistic understanding.

## Data collection

Data collection, spanning 2021–2023, began alongside community engagement and development activities. Aligned with our emic-etic research strategy, three modalities was used to capture the evolution of community engagement in the two compassionate communities and understand the influence of local contexts on community engagement processes, outcomes, and sustainability:

### Logbook (bridge and inside researchers)

Logbooks, recorded in Excel sheets, tracked project activities, documenting engagement (who was engaged, in what activities, and with whom) and identifying barriers and facilitators to community engagement. From 2022 to 2023, data was collected through bimonthly meetings between the *bridge* and *inside* researchers (CL, LJ)—community engagement facilitators—in Centre-Sud and West Island. During these meetings, the facilitators’ agendas systematically recorded all engaged partners and daily activities in an Excel sheet (one per site). The logbook data, gathered as ‘indirect participant observation’, tracked the project’s history and implementation. Logbook meetings further enabled the bridge researcher and community engagement facilitators to reflect on project progress, address challenges and biases, and refine action strategies.

### Semistructured interviews (bridge and inside researchers)

Semistructured interviews were conducted by the *bridge* researcher with various participants involved in the development of the Centre-Sud or West Island Compassionate Community. Participant selection used a purposive sampling strategy, adjusting numbers as the project evolved (e.g., staff turnover, arrival of new partners) and ensuring interviewees were knowledgeable about compassionate communities. Interviews with facilitators and the project leader (‘*inside*’ researchers) were conducted annually (2021–2023). Interviews with community partners were conducted in the final year of the project (2023). Involvement in the development of either compassionate community was the sole inclusion criterion for participation.

### Participant observation (bridge researcher)

Participant observation was conducted by the bridge researcher (E.L.), who was embedded within the mobilization, engagement, and community development activities (e.g., governance meetings, co-design programs, citizen forums). This approach bridged fieldwork and research, enabling the bridge researcher to contribute to the project’s development, support the action-research team, and gather data for knowledge production. *Inside* researchers (C.L., L.J., S.D., D.W.) invited the *bridge* researcher to meetings and activities involving community engagement and development (online or in person). The bridge researcher introduced herself to participants, describing her role and explaining the goal of participant observation. Participants could refuse at any time, but no one took this option. Observations focused on engagement relationships and dynamics, community partners’ needs, concerns, issues, visions, and ideas.

### Ethical considerations and reflexive practice

Navigating fieldwork during the COVID-19 pandemic presented unique ethical challenges. With an initial year of online-only community engagement due to public health restrictions (2021–2022), the informal interactions common to ethnography were limited, making trust the central ethical priority. To build and maintain trust, the bridge researcher focused on reciprocal practices, such as sharing observation notes and research tools like evaluation questionnaires after online meetings. Once in-person work resumed, this was complemented by actively participating with on-site activities (e.g., distributing meals to homeless people, or practical help in preparing activities). Ethical oversight was maintained through multifaceted reflexive practice. This included integrating reflexive notes within ethnographic fieldnotes,^12^ alongside regular logbook and team meetings, to critically assess positionality, its influence on the research, and any potential biases.

### Data collection summary

We collected data using participant observation, semi-structured interviews, and detailed logbooks to capture engagement and implementation processes at both sites. The greater number of participant observation hours in Centre-Sud reflects the later start of fieldwork in West Island due to contextual delays. Table 1 provides a comprehensive breakdown of all data collection activities and participant numbers for each site.

**Table 1.** Summary of the data collection methods and participants for each research settings (Centre-Sud and West Island).

Data collection methods	Centre-Sud (*n* = time, setting, participants, meetings, and interviews)	West Island (*n* = time, setting, participants, meetings, and interviews)	Total Centre-Sud and West Island
Participant observation (2021–2023)	63 h (2021–2023): over 200 community members were observed	21 h (2022–2023): nearly 100 community members were observed	Total of 84 h of participant observation with over 300 community members
Semi-structured interviews (2021–2023)	Interviews *n* = 17 with 14 participants: community engagement facilitators (*n* = 3); and community partners (*n* = 11) Length: 17 h with an average of 56.4 min	Interviews *n* = 9 with 8 participants: community engagement coordinator (*n* = 2); project leader (*n* = 1 - 2 interview); and community partners (*n* = 5) Length: 10 h with an average of 66 min	Total interviews: *n* = 26 with 22 participants Length: 27 h of interviews with an average of 64 mins
Logbook data inputs (2022–2023)	646 inputs Number of meetings: 41 Total length of sessions: 82 h	528 inputs Number of meetings: 35 Total length of sessions: 70 h	Total of 1174 inputs Number of meetings: 76 Total length of sessions: 152 h
Total multi-site ethnography	162 h	101 h	263 h

## Data analysis

The comparative analysis of the Centre-Sud and West Island Compassionate Communities drew on data from logbooks, participant observation, and interviews to examine local contexts, engagement processes, partners involved, activities implemented, populations reached, leadership, governance, and immediate outcomes. By exploring variations between the two communities, the analysis sought to uncover the mechanisms driving community engagement and to empirically inform new theoretical perspectives. The data analysis, both qualitative and descriptive, aimed to comparatively study the initiation and evolution of community engagement processes, as well as their influence on the development of both compassionate communities. An intracase analysis first allowed for understanding the evolution of each process, and then an intercase analysis highlighted similarities and differences between the two settings.^13^ All qualitative data was organized using QSR International NVivo 12 software, and pseudonyms were assigned to participants. Logbook data have been compiled and analyzed using Google Sheet.

**Figure 1.**
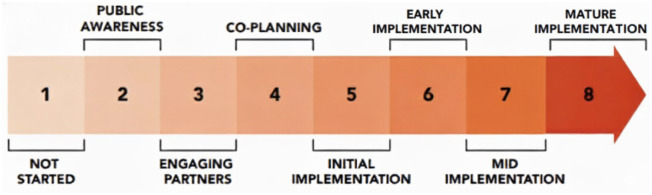
Canadian compassionate communities’ stages of development.^18^ Source: It is used with permission of the authors: Pallium Canada, BC Centre for Palliative Care, Hospice Palliative Care Ontario (2022).

The analysis was guided by two frameworks: the ‘Ecology of Engagement’^14^ and the ‘Compassionate Communities’ Stages of Development’^15^ (see Figure 1), which provided a systematized coding framework. While the stages of development are presented linearly, it is important to acknowledge the iterative nature of this process, with multiple back and forth movement between stages. The analysis was then guided by a thematic lens approach, as conceptualized by DeGloma and Papadantonakis.^16^ This approach uses a broad, socially significant theme, in this case ‘community engagement’, as the primary interpretive lens for the comparative analysis.^16,17^ This facilitated an iterative coding process involving both deductive coding, applying the two established frameworks, and inductive coding, using the thematic lens to identify emergent patterns.

Rigor and reliability were ensured through a multifaceted triangulation strategy. This involved comparing findings across data sources (interviews, logbooks, and participant observation notes) and triangulating perspectives. The latter involved validating the ‘bridge’ researcher’s interpretation with ‘inside’ and ‘outside’ researchers, after which the consolidated team findings were cross-validated with research participants via member checking. To further strengthen this process, artificial intelligence (AI) was used as a supplementary validation tool.

AI is increasingly recognized as a tool for managing and analyzing qualitative data.^19,20^ For this study, Gemini was chosen for its multilingual training, which is reportedly well-suited to the contextual nuances of distinct language variants^21^ such as Quebec French,^22^ our data language. The initial analysis, including all coding, categorization, and preliminary results, was conducted by the first author, who also acted as the ‘bridge’ researcher. To enhance objectivity given this embedded role, Gemini AI was used as a supplementary tool, a practice supported by literature on the use of AI in qualitative analysis.^19^ The AI’s role was confined to two specific tasks: (1) assisting in the identification of high-level and nuanced patterns from the preliminary findings, and (2) refining the English translation of quotes and improving the manuscript’s overall language quality. To ensure rigorous human oversight, the first author critically reviewed all AI-generated suggestions and retained full authority over all final analytical decisions.

## Results

This section presents key findings from our comparative ethnographic study. We begin with the communities’ definitions of engagement and compassionate communities, followed by an analysis of their distinct cultural contexts. Then, we contrast their initiation and engagement processes. Finally, we examine how development trajectories, engagement relationships, and leadership strategies influenced the communities’ development.

### Community voices in action: Defining community engagement and compassionate communities together

Aligned with our participatory approach to research, we made sure to include how participants defined community engagement and what compassionate community means to them in the semi-structured interviews with community members from Centre-Sud (*n* = 11) and West Island (*n* = 5). Community engagement has been defined very similarly by both communities as follows:

a collaborative and participatory process that involves diverse stakeholders working together to address both immediate needs and underlying root causes of community issues, improve well-being, and foster positive social change through concrete actions.

Participants, to whom we have assigned pseudonyms, mentioned that the motivations for community engagement are driven by a desire for social justice and equity, through advocating for the rights and well-being of vulnerable populations, promoting collective thinking and concerted action to develop local solutions to meet the needs. Mélanie, from Centre-Sud, shared:

Well, for me, I see community engagement as a response to suffering, particularly suffering caused by social injustice. Whether I experience it directly or witness it in others, the call to action is the same. It’s a movement of the heart, a recognition of our shared humanity.

Nathalie, from West Island, stressed the importance of concerted action to meet different end-of-life needs:

Well, often it’s gaps in the public health system, but it can also be, for example, we offer a program for bereaved toddlers. So there can be gaps more at the level of school boards and what they can offer to bereaved children, for instance. So, it’s about meeting needs and also filling existing gaps.

Centre-Sud and West Island both defined compassionate communities as a dynamic and collaborative network of individuals, community organizations, and civic institutions committed to

1. Promoting the holistic well-being of people facing serious illness, loss, end-of-life, death, and grief;
2. Fostering inclusion, social justice, and equity by ensuring that everyone has access to appropriate support and care;
3. Supporting the autonomy and independence of individuals by allowing them to make informed decisions and actively participate in community life;
4. Reflecting and acting collectively to address the community’s needs regarding loss, end-of-life, death, and grief;
5. Creating a strong support network by connecting people to resources, services, and individuals who can help them.

A participant from Centre-Sud introduced a unique experiential dimension to the definition of a compassionate community. Maude, a social worker in homelessness, challenged the research team to move beyond theoretical definitions. She underscored that a compassionate community is not merely an idea or a concept, but a lived experience that evokes a feeling of safety and support, where individuals feel empowered to try new things and explore possibilities:

For me, a compassionate community is more of a feeling. It’s like a safe space where you can say, ‘OK, we can try things, we can attempt things’. I see it like that for myself and for my clients.

This perspective adds a layer of emotional depth to the definition, highlighting the importance, for any compassionate community, to create a safe and inclusive environment where individuals feel valued, listened to, and where they can experiment with solutions by themselves.

### Comparing distinct cultural contexts

Despite shared vision and goals, West Island and Centre-Sud forged different paths in developing compassionate communities. This comparative analysis reveals that despite commonalities, local contexts shaped distinct engagement processes, leading to notable differences in development, outcomes, leadership, and sustainability. These insightful findings underscore the importance of understanding both shared patterns and contextual variations. The following section will first explore how local contexts influence community engagement processes, and then, we will examine the resulting variations in development, outcomes, leadership, and sustainability.

Montréal’s history is marked by its origins as a French settlement and a pivotal site in the British conquest of New France in 1759. This historical foundation has led to the development of diverse linguistic and cultural communities on the Montréal Island, creating the contrasting local contexts that are central to this ethnographic comparative study.

Centre-Sud, a neighborhood within the Ville-Marie borough in downtown Montréal, has over 40,000 residents within a 4.7 km^2^ area, and is historically a working-class district.^23^ Centre-Sud is characterized by its diverse social fabric, housing several vulnerable or marginalized populations, including the LGBTQ+ community, individuals experiencing homelessness and social precarity, refugees, drug users, and sex workers. On the other hand, West Island is a large suburban territory with over 266,000 residents spread across 144.4 km^2^.^24^ The West Island is predominantly English-speaking, though this population is a minority on the Island of Montréal, and tends to be older and wealthier than the average Montréal resident. Annex 1 provides a comparative local context table detailing the sociodemographic profile, environmental scan, asset mapping, and distinct historical features of both communities.

### Initiating compassionate communities: From early steps to community engagement processes (stages 1–4)

It is crucial to emphasize the significance of the preparatory phase (stages 1 and 2) before delving into the comparative community engagement processes (stages 3–4) and implementation trajectories (stages 5–8). This phase, while under-reported in the literature, involves understanding the unique local context and assessing the community’s readiness for engagement. These factors are pivotal for the success of any compassionate community initiative. The following section details stages 1–4 (see Figure 1), outlining the early steps involved in initiating community engagement processes within each compassionate community.

In 2020, guided by an asset-based community development framework,^25^ we conducted environmental scans and asset mapping within the Centre-Sud and West Island communities. Environmental scans compiled demographic, health, and social data, culminating in comprehensive community profiles, which were further enriched with historical and cultural context (see Annex 1 for detailed comparison of local contexts). Asset mapping identifies existing resources, strengths, and potential partners, directly informing the development of tailored strategies for mobilizing and engaging community leaders and champions within these territories. These initial findings serve as the foundation for ongoing iterative development cycles, ensuring the continuous adaptation and refinement of community engagement strategies throughout the project.

#### West Island initiation and community engagement process (stages 2–3)

In December 2019, the community mobilization efforts in the West Island began with a Town Hall meeting at the Teresa Dellar Palliative Care Residence. The meeting brought together 22 representatives from various community and civic organizations. In addition, the community engagement coordinator met individually with more than 20 representatives of community organizations and civic institutions (i.e., City Council) to raise awareness about the Compassionate Community approach, and engage them in identifying needs and solutions. Awareness-raising and mobilization activities took place from March 2020 to May 2022, at the height of the pandemic. Consequently, the needs assessment strategy was adjusted to target caregivers using an online questionnaire (*n* = 21).

Throughout these 2 years (December 2019 to May 2022), community mobilization and engagement capacities were significantly diminished in the West Island. As Sarah, an executive director of a local community organization, explained:

I think there are eighty community organizations on the West Island. Then, we were just twenty that were still open throughout the pandemic.

This situation significantly impeded early community engagement in the project’s early stages. Consequently, the development strategy was adjusted to supplement the community-led approach with an institutionally led orientation. This multistrategy approach further facilitated partnerships for developing compassionate programs.

In the West Island, the results point to three facilitators of community engagement: (1) engaging with individuals driven by a strong sense of duty; (2) being proactive in building trust-based relationships (recognizing expertise of the community); (3) and reciprocal relationships that foster an inclusive and collaborative environment. A shared language, common vision, and goals further enhance engagement. Patricia, Director of a nonprofit home support organization, stated:

They [Palliative Care Residence] recently asked to be part of the Seniors’ Table. Before that, they were really excluded from other non-profit organizations, and they did everything internally. So, like I said, if this project can open the door so that all the other organizations in the West Island, which are not as big as the Residence, are recognized for the work they do. Sure, if there’s funding, cool, but just to say that they are not alone in caring for people at the end of life.

Michael, a primary care community worker, emphasized the role of reciprocal relationships in building trust:

They [community members] will share information with us, because they trust us, because it’s a relationship that goes both ways. (. . .) These are not relationships that were created yesterday. These are relationships from many times we came to lend a hand. So there is already this credibility.

These examples illustrate how effective communication, built on transparency, and consistent engagement based on a shared vision foster trust and reciprocity, cultivating collaborative environments where diverse stakeholders feel valued and work together toward a common goal.

#### Centre-Sud initiation and community engagement process (stages 2–3)

During fall 2020, two community engagement facilitators in Centre-Sud individually connected with 68 community organizations to raise awareness about end-of-life, loss, death, and grief, introducing them to the concept of compassionate communities. From February to May 2021, the coordinators facilitated eleven meetings, organized by sector (*n* = 5) and target population (*n* = 6), to collaboratively identify needs and potential solutions with representatives from these organizations. During these meetings, participants were asked to share their experiences with death both within their organizations and in their personal lives. This open invitation to discuss death, sparked community engagement and laid the foundation for a compassionate community in Centre-Sud, built upon shared experiences of end-of-life, loss, death, and grief. The community engagement process was thus centered on shared needs and resource pooling, characterized by a grassroots, community-led approach that promoted ownership.

Community engagement facilitators have been able to build trust through a humble attitude (recognizing the community’s expertise in end-of-life and grief), active listening, while our participatory research approach relied on reciprocal relationships that support the development and implementation of compassionate community. Five contextual factors fostered community engagement in Centre-Sud: the pandemic crisis, shared experiences and values, strong relationships, knowledge sharing, and supportive research practices (organizational support). Despite these positive factors, challenges such as workforce shortages and communication barriers were present, but these had little effect on the overall community engagement process.

At both sites, a change of community engagement facilitators occurred within a year (2021–2022). This pivotal moment, which initially threatened to disrupt project momentum, became a valuable learning experience. It highlighted the importance of selecting coordinators with deep roots and knowledge of the community ecosystem. The new coordinators (C.L., L.J.), well-known community workers with decades of experience in their respective communities, fostered greater engagement, trust, and collaboration. This enabled us to co-create and experiment with projects, reaching partners who were previously inaccessible to our action-research team or community outsiders.

### Contrasting paths to community engagement

**Graph 1.**
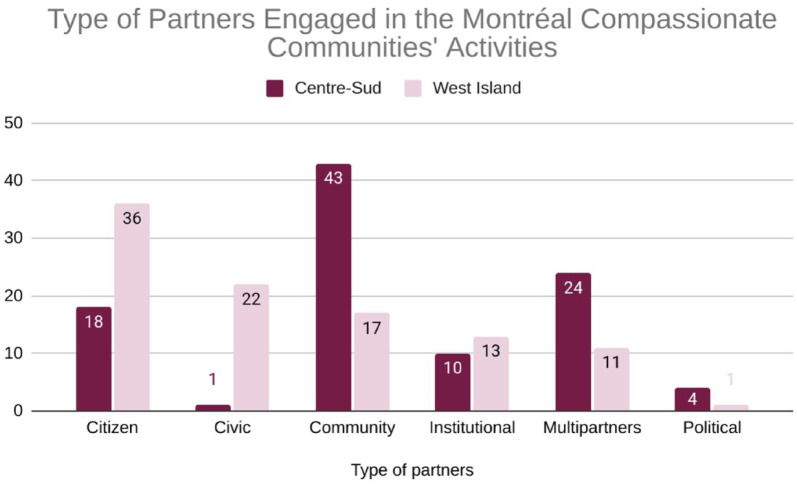
Comparison of types of partners engaged in compassionate community activities in Centre-Sud and West Island (2021–2023).

The contrasting engagement strategies in Centre-Sud and West Island are clearly illustrated by the types of partners each initiative engaged, as shown in Graph 1. For this analysis, each logbook activity (e.g., meeting, workshop, follow-up) was categorized by partner type, defined as: *Citizen* (i.e., volunteer); *Civic* (i.e., municipalities, workplaces); *Community* (i.e., local organization representative); *Institutional* (i.e., health and social services, local police); *Political* (i.e., elected official); and *Multipartner*, which reflects activities involving multiple partner types.

Graph 1 highlights distinct partner engagement patterns that reflect two different strategic approaches. West Island’s (*n* = 329) higher proportion of *Citizen* (36.17%) and *Civic* (22.19%) engagement demonstrates its institutionally led strategy focused on raising public awareness and encouraging individual participation. Conversely, Centre-Sud’s (*n* = 509) emphasis on *Community* (43.42%) and *Multipartners* (24%) activities reflects its grassroots strategy, centered on building a resilient network of inter-organizational collaboration. While institutional engagement was relatively similar, the variations in other partner types underscore how different engagement strategies were shaped by local contextual factors, including the COVID-19 pandemic.

In Centre-Sud, a grassroots, community-led strategy, focused on shared experiences of mortality, fostered trust, and cohesion, leading to higher engagement and ownership despite the COVID-19 pandemic. The pandemic paradoxically catalyzed community mobilization around mortality and grief. Jean-Baptiste, a community support program director, noted:

I felt like there was a really fitting connection between the project’s values and the pandemic context (. . .) It was the perfect time for this project to come along, with everything we were noticing internally. I saw an opportunity and went for it wholeheartedly.

This shared sense of purpose was further bolstered by the neighborhood’s rich tradition of social activism and deeply ingrained culture of collaboration. A bottom-up, community-led, and contextually attuned approach was associated with successful community engagement in Centre-Sud. Although the facilitators’ specific contributions were highlighted in this study, they will be detailed further in our next publication.

West Island’s multistrategy approach to engagement, initiated earlier, faced challenges due to pandemic restrictions and contextual factors. With its aging population, a large proportion of community support funding is dedicated to seniors. This fact, coupled with limited, nonrecurring funding, fostered competition, and distrust within the community. As Sarah, a Volunteer Center Director, explained:

It caused a bit of competition. Instead of giving funding to each organization where they’ve already justified [their impacts], they required accountability reports. It’s people who don’t manage things properly. Why not give us extra money without having to make [funding] applications each year.

Patricia, senior’s home support director, highlighted the hesitancy of smaller organizations to engage with the Palliative Care Residence, citing past experiences of resource competition and program duplication:

When there’s a big entity, like the Palliative Care Residence that keeps getting government funding for programs that are technically already in place by other organizations. At some point, the organizations start to get fed up, you know. So yeah, there was a coldness in the West Island [community] towards the Teresa Dellar Palliative Care Residence.

These challenges illustrate how pandemics and funding structures can impede engagement, especially with preexisting scarcity and distrust.

### Comparing development trajectories (stages 5–8)

**Graph 2.**
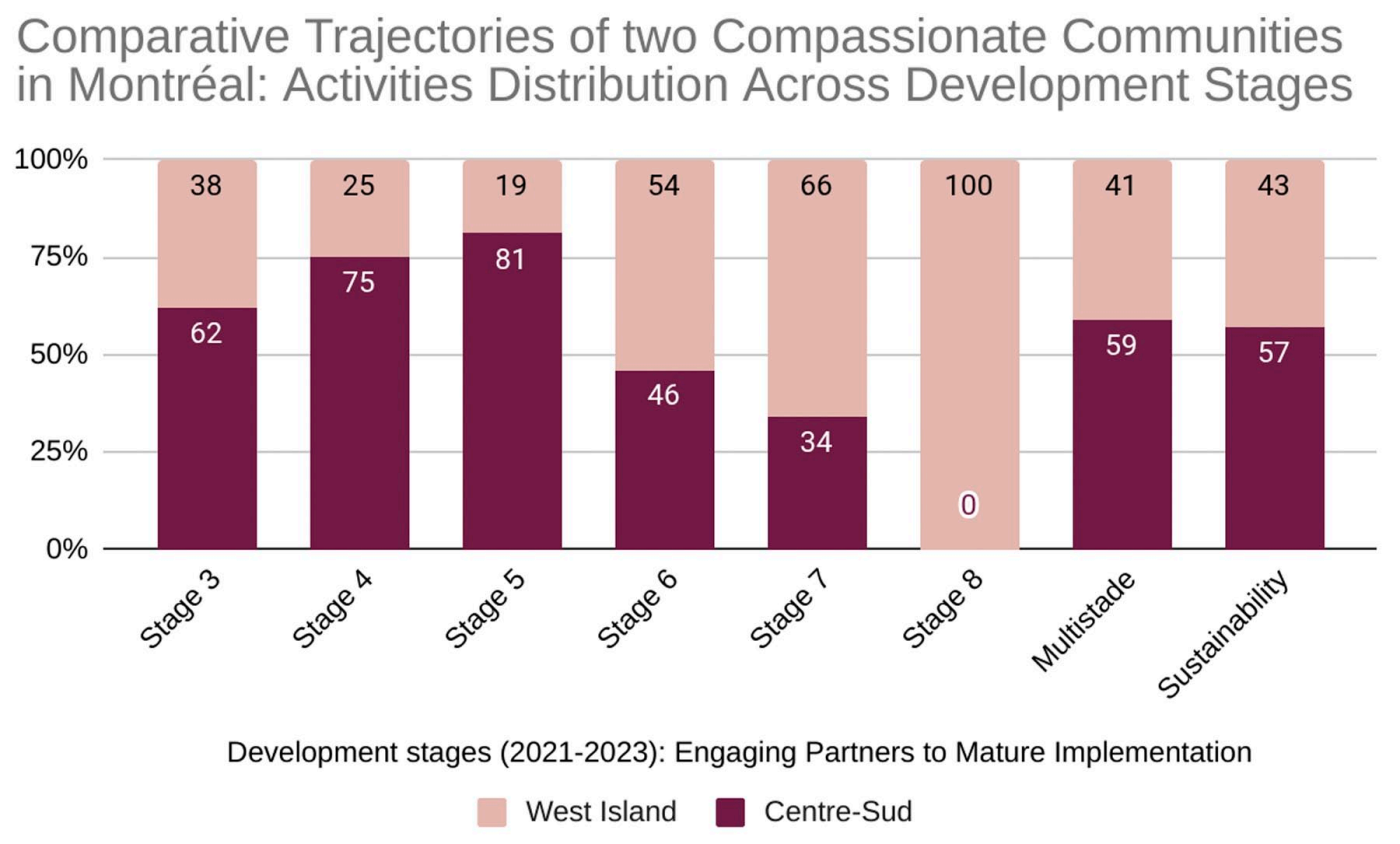
Comparative trajectories of two compassionate communities in Montréal: activity distribution across development stages. 3: Engaging partners; 4: Co-planning; 5: Initial implementation; 6: Early implementation; 7: Mid implementation; 8: Mature implementation.

The different engagement strategies of the two communities resulted in distinct development trajectories, with each investing its efforts at different stages of the process, as illustrated in Graph 2.

As the data show, the two communities prioritized their efforts differently. Centre-Sud, with a higher total number of activities (*n* = 637), concentrated its work in the earlier co-planning stages (stages 3–5), prioritizing the development of trust and collaborative relationships before scaling programs. Conversely, West Island (*n* = 467) focused its activities on the later implementation stages (stages 5–8). This reflects the pragmatic shift by the Palliative Care Residence to ensure tangible programs were established when broader engagement was difficult. This highlights a foundational difference: Centre-Sud invested in building community ownership first, while West Island focused on delivering services to address immediate needs.

#### Who is engaged and in what kind of activity?

Logbook data analysis revealed the diverse ways West Island and Centre-Sud Compassionate Communities engaged with partners and how it evolved over time. Using the Ecology of Engagement framework,^13^ we categorized activities across eight development stages. For the following analysis, engagement relationships were categorized as follows:

- *Bonding*: Connections *within* groups sharing similar identities, fostering a sense of belonging and trust (e.g., community organizations working together);
- *Bridging*: Connections *between* groups with different backgrounds, identities and interests, promoting collaboration and understanding (e.g., a partnership between a community center and a Palliative Care Residence);
- *Linking*: Connections between individuals or groups *across* different levels of power, facilitating access to resources and equitable decision-making (e.g., community partners collaborating with city officials on community support initiatives).

‘Multistage’ refers to activities where bonding, bridging, and linking relationships occur simultaneously within the same activity. ‘Sustainability’ reflects activities contributing to long-term initiatives, not a distinct development stage. The resulting graphs illustrate distinct trajectories and highlight how these relationships shift throughout both compassionate communities’ development.

**Graph 3.**
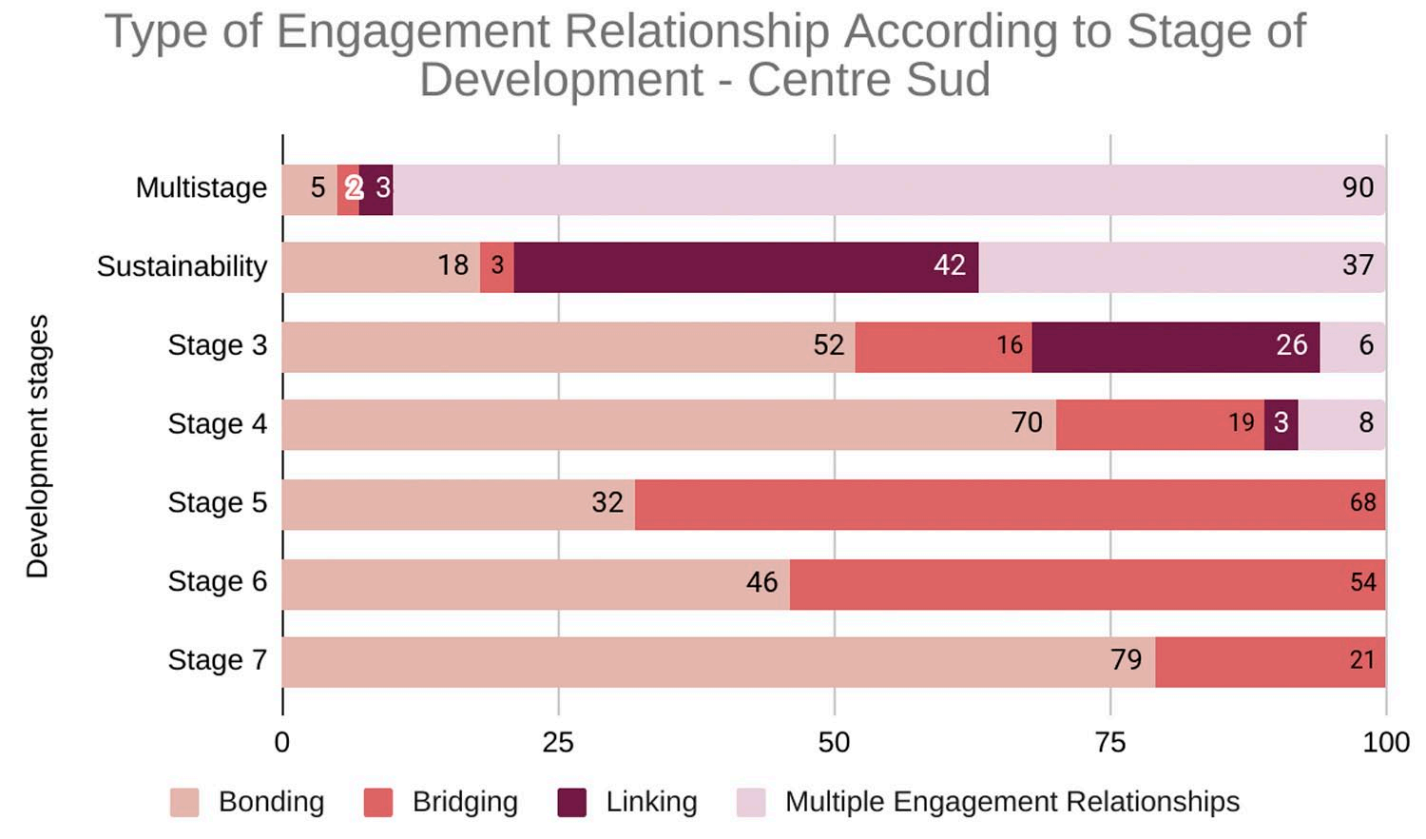
Engagement relationships throughout the development of Centre-Sud Compassionate Community (*n* = 515). 3: Engaging partners; 4: Co-planning; 5: Initial implementation; 6: Early implementation; 7: Mid implementation.

Graph 3 illustrates engagement patterns across development stages in Centre-Sud (*n* = 515).

Centre-Sud initially focused on bonding within community groups during engagement and co-creation (stages 3–4), evidenced by a high number of bonding relationships during these phases. Specifically, stage 4 shows a significant concentration of bonding relationships. As the project progressed, there was a notable shift toward bridging relationships in stage 5, indicating increased collaboration with external partners. The ‘Sustainability’ stage demonstrates a unique pattern with a high number of linking relationships, suggesting efforts to connect with resources and power structures for long-term impact. Additionally, a significant proportion of activities were categorized as ‘Multistage’, highlighting the complexity and interconnectedness of development efforts. In the following graph, engagement relationships are categorized as follows: *Bonding* (connecting similar groups), *Bridging* (connecting different groups), and *Linking* (connecting to power/resources). The data show a progression from building internal trust and support (bonding) to fostering wider collaborations (bridging) and securing long-term resources (linking) as displayed in Graph 3.

**Graph 4.**
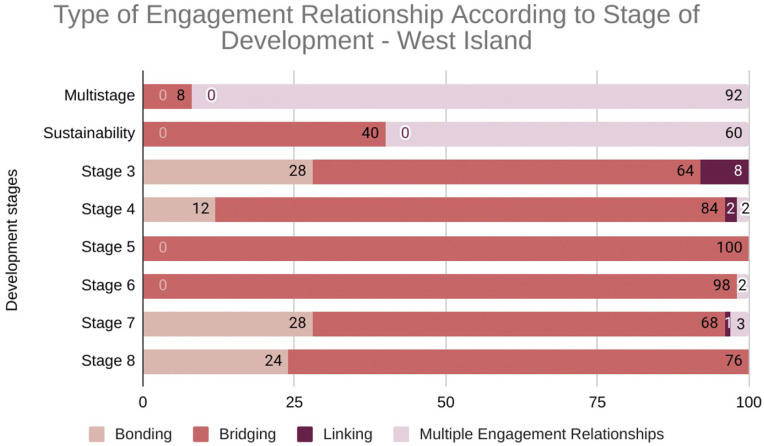
Engagement relationships trajectories throughout the development of West Island Compassionate Community (*n* = 330). 3: Engaging partners; 4: Co-planning; 5: Initial implementation; 6: Early implementation; 7: Mid implementation; 8: Mature implementation.

Graph 4 illustrates engagement patterns across development stages in West Island (*n* = 330).

West Island primarily relied on bridging relationships throughout its development, indicating a strong emphasis on collaboration between palliative care professionals and the wider community. This is particularly evident in stages 4, 6, and 7, which show a high concentration of bridging relationships. Bonding relationships were less prominent, indicating a different approach to community support. Linking relationships were minimal throughout the West Island’s development, indicating less focus on connecting with external resources and power structures. The data suggest a consistent focus on building external partnerships (bridging) as the primary strategy for community engagement.

Graphs 3 and 4 reveal distinct engagement patterns. Centre-Sud evolved from bonding to bridging and linking, while West Island consistently emphasized bridging. Both initiated linking at stage 3, but these connections declined in West Island, remaining crucial for Centre-Sud’s sustainability. This divergence reflects evolving community needs and highlights the importance of context-specific strategies. Analyzing engagement types in sustainability activities and the proportion of multistage activities provides insights into long-term impact. For instance, high ‘multistage’ activity with strong linking relationships suggests a community is effectively building on diverse social capital and engaging with power structures to ensure long-term success.

#### Leadership and sustainability

We observed that intersectoral collaboration thrives when a dedicated individual champions the initiative within community organizations or healthcare institutions. This individual mobilizes their social environment by sharing knowledge about death and dying. However, this reliance poses a risk of knowledge loss if the champion departs. To mitigate this, documenting processes, training others, and establishing sustainable support structures are crucial. While sustainability was a key concern from the outset, our study found that evolving leadership aligned with community engagement and implementation strategies to ensure long-term viability. For instance, when research funding ended in December 2023, the Centre-Sud community incorporated as a nonprofit, ‘*Communauté Compatissante Montréal*’, to continue development activities and to seek funding on their own. Conversely, the Palliative Care Residence in the West Island community retained their community engagement facilitator in a permanent role to maintain momentum and pursue her work. These diverse approaches underscore how leadership and sustainability are intertwined with engagement strategies and implementation trajectories.

## Discussion

This study examined community engagement in developing compassionate communities within two culturally diverse Montréal neighborhoods. Such detailed empirical research is crucial to fill a significant evidence gap as recent reviews show that community engagement is infrequently and poorly reported in the literature.^2–4,25^ Our comparative analysis revealed distinct approaches, highlighting the influence of context and leadership. Centre-Sud’s grassroots, community-led approach fostered trust and collaboration, allowing it to thrive despite pandemic restrictions. In contrast, West Island’s professionally driven, multistrategy approach encountered challenges in fostering collective action due to resource limitations and preexisting community distrust, which were exacerbated by the pandemic.

Despite these differences, both communities demonstrated a strong commitment to addressing the holistic needs of individuals facing serious illness, end-of-life, death, and grief. Their experiences emphasize the importance of adaptability, responsiveness to context, and recognizing existing community strengths in developing and sustaining compassionate communities. Ultimately, this study reveals that diverse paths can lead to compassionate communities. While both communities possess inherent strengths and resources, their development trajectories differed significantly due to contextual factors and leadership approaches. This finding is consistent with a comparative study of initiatives in Italy and the United Kingdom, which found that the nature of an initiative’s promoter (e.g., public vs philanthropic) and the national context (e.g., social care vs healthcare-oriented) significantly shape the features of community engagement.^26^

These findings have important implications for developing and sustaining compassionate communities. First, they underscore the importance of understanding and responding to each community’s unique context. Top-down approaches may be appropriate in certain contexts, but seem less effective in communities characterized by distrust toward institutions, or limited resources, particularly during crises. This finding is strongly supported by reviews of place-based initiatives. These reviews identify power dynamics and a lack of trust as the most significant barriers to engagement. Conversely, they cite establishing trust—a process requiring long-term commitment—as the single most critical enabler for success.^4,27^ Centre-Sud’s success demonstrates this principle, offering a key lesson for practitioners: budget significant time for foundational, trust-building activities before other outcomes can be expected.

Second, our research highlights the need to identify and collaborate with existing community leaders and champions. By leveraging the strengths and recognizing expertise of individuals and organizations already engaged in supporting those facing serious illness and loss, we can avoid duplication of services and promote more effective collaboration. However, effective collaboration is often hindered by policy-level barriers. Our finding that competition and distrust in West Island were linked to funding structures aligns with broader research identifying short-term, inflexible funding as a major obstacle to sustainable community initiatives.^25,28^ This points to a crucial policy implication: the need for long-term funding cycles that support the time it takes to build relationships and allow for meaningful evaluation.^27^ Moreover, our findings showed that successful community engagement also relies on the personal qualities and experience of the community engagement facilitators. Technical skills are important, but active listening, empathy, humility, and strong community ties—meaning coordinators who come from and have a deep understanding of the community—are essential for building trust, fostering collaboration, and achieving meaningful outcomes.

### Key levers of engagement: Building trust and navigating power

The divergence in development trajectories—prioritizing programs versus relationships—offers a critical insight into navigating community trust and power. Centre-Sud’s strategy of investing time in relational foundations before implementation directly aligns with broader research confirming that establishing trust is an essential precursor to any successful community-led initiative, a process requiring significant time and commitment.^27,28^ In contrast, the West Island’s approach demonstrates a necessary adaptation and flexibility to navigate its specific contextual barriers.

Our analysis using the Ecology of Engagement framework further illuminates these context-specific strategies. West Island consistently relied on bridging relationships between palliative care professionals and the wider community, while Centre-Sud initially focused on bonding within community groups before expanding its reach. The difficulty in establishing bridging capital in West Island, in contrast to its success in Centre-Sud, mirrors findings from other complex systems where weak cross-sector connections are often linked to historical power imbalances and a lack of shared vision.^28^ Ultimately, evolving leadership played a crucial role in ensuring long-term viability in both contexts. Centre-Sud achieved sustainability by incorporating as an independent nonprofit, while West Island did so by retaining their facilitator in a permanent, institutionally supported role. These diverse approaches underscore that sustainability, leadership, and engagement strategies are inextricably linked.

### Beyond engagement: The transformative potential of shared power

Our research highlights the transformative potential of community engagement in developing compassionate communities. By emphasizing shared leadership and power between researchers, community partners, health, and social services, participatory approaches promote social participation and empower communities to take collective responsibility for caring for those who are seriously ill, dying, or grieving. This aligns with calls to reorient health services by rebalancing power—a significant challenge, particularly when integrating community approaches into traditionally hierarchical settings like hospitals.^29,30^ This collaborative approach challenges the traditional medical model of palliative care, and has the potential to lead to profound cultural transformations in how we interact with illness, death, dying, and grief.

Sharing responsibility necessitates sharing power across the healthcare system, communities, families, and individuals. This collaborative approach recognizes that everyone’s role is essential in developing community-based support systems that complement palliative and end-of-life care. The partnerships between academic researchers and communities proved invaluable in highlighting the contributions of all stakeholders. Notably, community and non-profit organizations that had not previously identified with the compassionate community model began to see its relevance to their work. By facilitating intersectoral collaboration, supporting social participation, and empowering communities, such a participatory approach to research actively contributes to the compassionate community movement.

### Implications for future research

Mapping this broader care ecosystem at the micro and meso levels, regardless of the theoretical lens used (e.g., community engagement,^14^ caring neighborhood model,^31^ circle of care concept^32^), allows research to make visible the daily work and care provided by caregivers, community organizations, and citizens. This mapping process helped identify community leaders, players, and champions with whom to engage. These individuals, dedicated to improving the living and dying conditions of their fellow human beings, are the foundation upon which compassionate communities and other public health initiatives related to death and dying can exist.

Building on this, future research should prioritize several key areas. First, a deeper investigation is needed into how different engagement types (e.g., bonding, bridging, linking) influence the specific outcomes and sustainability in various settings. Second, research should look beyond engagement strategies to assess local contexts and the underlying capacity of the participating organizations. Drawing on Näsman et al.’s^33^ postulate that sociostructural resources predict associational participation, future studies could explore whether organizational capacity (e.g., funding, staffing, social capital) is a key determinant of success, particularly in resource-constrained contexts. Finally, our study observed that in both communities, exchanges about experiences of end-of-life, death, and loss fostered mutual support and a sense of shared humanity. Further research examining social changes (e.g., community support, death literacy) and cultural transformations (e.g., breaking the taboo with shared narratives that bring new meanings to death) is essential to explore the long-term impact of these initiatives and identify best practices to support their sustainable development. Answering these questions will provide crucial evidence for developing and evaluating compassionate communities across diverse cultural and economic landscapes.

### Limitations

This study has several limitations. Findings may have limited generalizability due to the focus on two specific Montréal communities, and the results were influenced by the COVID-19 pandemic and the inherent challenges of comparing two culturally distinct contexts. Several potential sources of bias should also be noted. The staggered start of fieldwork resulted in unequal participant observation data between the two sites, which may have affected the comparative analysis. Furthermore, we acknowledge a potential for self-selection bias, as individuals who chose to participate in interviews and activities may have held stronger or more positive views than the wider community. While the use of AI as a supplementary tool enhanced objectivity, we recognize its inherent limitations. The risk of the AI missing nuances or generating misinterpretations was proactively minimized by our research design, as it was only used on preliminary findings already generated by the ‘bridge’ researcher. Furthermore, the results were validated with participants through member checking to ensure the credibility and accuracy of the final interpretations. As a final safeguard, the first author critically reviewed all AI-generated outputs against the original data and retained full authority over all analytical decisions.

## Conclusion

This comparative study of two contrasting Montréal neighborhoods demonstrates that successful community engagement is fundamentally context-sensitive. Our findings reveal that while the community-led strategy in Centre-Sud thrived by fostering deep ownership and empowerment, the institutionally led model in West Island proved a necessary and viable alternative to navigate systemic barriers and establish a sustainable initiative.

Ultimately, this study contributes to the limited empirical literature by showing that success is not defined by a single approach, but by aligning the engagement strategy with a community’s unique sociocultural realities, which in turn shape its internal dynamics of trust, power, and resources. These findings offer valuable insights for practitioners and policymakers, underscoring the importance of context-sensitive strategies to build resilient compassionate communities that empower people to take collective responsibility to care for those facing serious illness, end-of-life, death, and grief.

## Supplemental Material

sj-docx-1-pcr-10.1177_26323524251400806 – Supplemental material for Community engagement in public health palliative care: A comparative ethnographic study of two culturally distinct compassionate communities in Canada Supplemental material, sj-docx-1-pcr-10.1177_26323524251400806 for Community engagement in public health palliative care: A comparative ethnographic study of two culturally distinct compassionate communities in Canada by Émilie Lessard, Isabelle Marcoux, Serge Daneault, Lise Jean, Cynthia Lapointe, Dale Weil, Ghislaine Rouly, Libby Sallnow, Allan Kellehear and Antoine Boivin in Palliative Care and Social Practice

## Appendix

**Annex 1.** Comparative features of Centre-Sud and West Island Compassionate Communities.

Features	Centre-Sud Compassionate Community	West Island Compassionate Community	Key differences and similarities
*Community size* Approximate population, geographic area	- Centre-Sud is a neighborhood spreading over 4.7 km² located downtown Montréal with a population over 40,000 (more than 15,000 people are expected over the next 10 years)	- The West Island of Montréal is a large suburban territory (including many cities and boroughs) which covers a large territory extending over 144.4 km² with a population over 266,000	*Comparing sizes and potential impact on initiatives* Centre-Sud is a relatively small area with over a hundred community organizations. This geographic proximity and the high concentration of organizations facilitate community mobilization and collaboration. In comparison, the West Island is more than 30 times the size of Centre-Sud. As a result, civic and community organizations are far apart, making community mobilization and collaboration more challenging
*Demographics* Age distribution, gender ratios, ethnic diversity, socioeconomic status, etc.	Compared to the total population of the City of Montréal (CDC Centre-Sud, 2023), Centre-Sud has: - a higher proportion of residents aged 15–44 and fewer children aged 0–14; - average household incomes remain lower as it is primarily composed of couples without children and single-person households; - gender ratio is 57% male and 43% female, which is consistently higher than Montréal average, because a majority of gay men live in the gay village at the heart of Centre-Sud	Compared to the total population of the City of Montréal (2022), West Island has: - a higher proportion of residents aged 65 and older; - average household incomes remain higher, and single-person households are mostly associated with immigrants and seniors	*Identifying variations or commonalities in demographics* *Commonalities*: • *High ethnic diversity:* All three areas exhibit high ethnic diversity, reflecting Montréal’s multicultural character *Variations*: • *Age distribution*: ○ Centre-Sud: Younger population (15–44 years old) with fewer children ○ West Island: Older population (65+ years old) ○ Montréal: More balanced age distribution • *Household composition*: ○ Centre-Sud: Primarily couples without children and single-person households ○ West Island: Higher proportion of single-person households (often associated with immigrants and seniors) ○ Montréal: More diverse mix of household compositions • *Gender ratio*: ○ Centre-Sud: More men than women, due to the gay village ○ West Island: Likely close to the Montréal average ○ Montréal: Slightly more women than men • *Socioeconomic status*: ○ Centre-Sud: Lower average household income ○ West Island: Higher average household income Montréal: Average income falls between Centre-Sud and West Island
*Community history/culture* Prevailing values, historical context, traditions, religious affiliations, etc.	- Over a century of social action tradition (influence of Catholic religion) - Historically poor working-class neighborhood - Vulnerable populations (homelessness, addictions, social isolation) - LGBTQ+ community settled in the neighborhood in 1982 (gay village) - Red light district	- Post-war era saw a significant shift from rural to suburban life, with the development of residential neighborhoods and shopping centers - Important economic hub, home to various industries and businesses - Multiculturalism: West Island has become increasingly diverse with immigrants from various parts of the world - Aging population and social isolation	*Cultural and historical influence on community interactions* The cultural contrast between French-Canadian and English-Canadian communities is rooted in historical events, notably the British conquest of New France in 1759. This pivotal moment led to tensions and power struggles over language, culture, and self-determination, shaping the relationship between the two communities. Religious traditions further contributed to this contrast, with Catholic charity historically focused on institutional support and Protestant charity emphasizing private individual support. This historical and cultural context has influenced community interactions and engagement in compassionate communities
*Existing social networks* Strength/overview of existing connections, community organizations, volunteer groups	- High concentration of community resources for diverse vulnerable population (homelessness, drug addiction, social insecurity, mental health, sex work, etc.)	- Many volunteer groups and community organizations dedicated to seniors population (caregivers, chronically ill, impaired or end-of-life seniors, social isolation) - Community resources for other vulnerable populations exists in proportion to the community’s needs	A wide variety of populations are served by community organizations in the Centre-Sud, while many West Island community resources are focused on the needs of their senior population. The West Island ‘homogeneity’ of the needs and the scarcity of resources available to community organizations leads to competition that can hinder collaboration
*Identified needs and population reach* (illness and dying trajectories) Specific areas where compassionate support is most needed (e.g., end-of-life, bereavement, caregivers)	- End-of-life support at home for isolated people; - Overdose bereavement support; - Intergenerational activity; - LGBTQ+ community seniors’ residences; - Support to practitioners in homelessness, addiction and poverty (social precariousness) confront to death on a daily basis at work intervenants en itinérance et dépendance confrontés à la mort tragique (surdoses, suicides, meurtres) = death literacy created within compassionate community	- Support at home for seniors and family caregivers, bereavement support, intergenerational activity, death literacy, peer support for seniors victims of fraud	*Pinpoint overlapping or unique needs for tailored interventions*: • *End-of-life support at home*: Both Centre-Sud and West Island have populations experiencing isolation and social precariousness, making home-based end-of-life care crucial. This is particularly important for individuals facing homelessness, addiction, or poverty • *Bereavement support*: Both communities likely experience various types of loss, including overdose-related deaths and the passing of loved ones due to illness or old age. Comprehensive bereavement support is needed, potentially with specialized services for overdose bereavement • *Death literacy and education*: Raising awareness about death and dying is relevant across both regions to foster open conversations, informed decision-making, and better support for those navigating end-of-life experiences • *Support for caregivers and families*: Family members and caregivers in both areas need resources and guidance to cope with the challenges of caring for dying loved ones or grieving their loss *Unique needs Centre-Sud*: • *Overdose bereavement support*: Given the higher prevalence of overdose deaths in Centre-Sud, specialized bereavement services tailored to this specific type of loss are essential • *LGBTQ+ community seniors’ residences*: The presence of LGBTQ+ seniors in Centre-Sud necessitates sensitive senior housing options and support services that cater to their unique needs and experiences • *Support for practitioners*: Professionals working with homelessness, addiction, and poverty in Centre-Sud face daily exposure to death and trauma. They would benefit greatly from specialized support services and death literacy training *Unique needs West Island*: • *Intergenerational activity*: Creating opportunities for intergenerational connection could be valuable, particularly considering the diverse cultural background of the population • *Peer support for seniors victims of fraud*: Addressing the specific issue of fraud targeting seniors through peer support groups and educational initiatives could be helpful
