# Supplementary material for: Community engagement in public health palliative care: A comparative ethnographic study of two culturally distinct compassionate communities in Canada
Source: Palliat Care Soc Pract. 2025 Dec 15;19:26323524251400806. doi: 10.1177/26323524251400806 (PMC12708986; doi:10.1177/26323524251400806)
Supplement: sj-docx-1-pcr-10.1177_26323524251400806 – Supplemental material for Community engagement in public health palliative care: A comparative ethnographic study of two culturally distinct compassionate communities in Canada [file sj-docx-1-pcr-10.1177_26323524251400806.docx]

### **Supplementary File 1. Standards for Reporting Qualitative Research (SRQR) Checklist**

| **#** | **Item** | **Manuscript Text and Location** |
| --- | --- | --- |
| **Title and Abstract** |  |  |
| 1 | Title | Community engagement in Public Health Palliative Care: A comparative ethnographic study of two culturally distinct compassionate communities in Canada (Title page) |
| 2 | Abstract | The full abstract text is provided in the manuscript (“p. 0”) and Plain Language Summary is provided on p.0-1 |
| **Introduction** |  |  |
| 3 | Problem Formulation | "Despite this global momentum and the fundamental role of community engagement in this model, empirical research remains underdeveloped, hindering evidence-based guidance for practitioners.^3–6^ Consequently, a critical gap persists in understanding how local contexts shape community engagement processes and outcomes within compassionate communities initiatives.  To address this gap, this comparative ethnographic study explores the interplay of local context and engagement processes in two culturally distinct compassionate communities in Montréal, Canada." (p. 1) |
| 4 | Purpose or Research Question | "By comparatively analyzing the processes in these two settings, we address the research question: *“How does community engagement evolve and influence the development, outcomes, and sustainability of compassionate community initiatives within diverse contexts?”* " (p. 1) |
| **Methods** |  |  |
| 5 | Qualitative Approach & Research Paradigm | "In this study, we used a comparative ethnographic design, which involves the in-depth, immersive study of people within their natural settings... Inspired by developmental evaluation, we documented the community engagement process and development trajectories prospectively in real-time through ethnographic methodology." (p. 2) |
| 6 | Researcher Characteristics & Reflexivity | "To trace the dynamic evolution and varied influences of community engagement, we adopted an emic-etic research approach. Drawing on ethnographic debates regarding researcher positionality—specifically 'emic' (insider) and 'etic' (outsider) perspectives, individual team members assumed specific roles within this framework, acting as 'insider,' 'outsider,' or 'bridge' researchers..." (p. 3). |
| 7 | Context | "To ensure the findings had broader applicability, two contrasting Montréal neighborhoods, Centre-Sud and West Island, were purposefully selected... because they have contrasting socio-cultural profiles (i.e. English/French cultures, richer/poorer and older/younger than average)..." (p. 2). *Further details are provided in the "Comparing Distinct Cultural Contexts" section* (p.8) and *Annex 1.* (p. 31) |
| 8 | Sampling Strategy | "Participant selection used a purposive sampling strategy, adjusting numbers as the project evolved (e.g., staff turnover, arrival of new partners) and ensuring interviewees were knowledgeable about compassionate communities. Involvement in the development of either compassionate community was the sole inclusion criterion for participation." (p. 4) |
| 9 | Ethical Issues | "This study received ethical approval from the research ethics board of the Centre hospitalier de l’Université de Montréal (#18.353)." (p. 4, 21).  “*Ethical Considerations and Reflexive Practice*  Navigating fieldwork during the COVID-19 pandemic presented unique ethical challenges. With an initial year of online-only community engagement due to public health restrictions (2021-2022), the informal interactions common to ethnography were limited, making trust the central ethical priority…”(p.4). *Declarations section:* No ethical issues to declare (p. 21) |
| 10 | Data Collection Methods | "Data collection, spanning from 2021 to 2023... three modalities were used to capture the evolution of community engagement...: Logbook... Semi-Structured interviews... Participant observation..." (p.3-4). Table 1. Summary of the data collection methods and participants for each research settings (Centre-Sud et West Island) (p.5) |
| 11 | Data Collection Instruments & Technologies | "Logbooks, recorded in Excel sheets to track project activities... Semi-structured interviews were conducted... Observations consisted of detailed field notes recorded during each event and activity." (p. 3-5) |
| 12 | Units of Study | Research Approach and Case Section “two contrasting Montréal neighborhoods, Centre-Sud and West Island, were purposefully selected... because they have contrasting socio-cultural profiles (i.e. English/French cultures, richer/poorer and older/younger than average)..." (p. 2). |
| 13 | Data Processing | "All qualitative data was organized using NVivo 12 software, and pseudonyms were assigned to participants. Logbook data have been compiled and analysed using Google Sheet... (p. 6) |
| 14 | Data Analysis | "The analysis was guided by two frameworks: the ‘Ecology of Engagement’^14^ and the ‘Compassionate Communities’ Stages of Development’^15^ (see Figure 1), which provided a systematized coding framework" ... This facilitated an iterative coding process involving both deductive coding, applying the two established frameworks, and inductive coding, using the thematic lens to identify emergent patterns." (p. 6) |
| 15 | Techniques to Enhance Trustworthiness | "Rigor and reliability were ensured through a multi-faceted triangulation strategy. This involved comparing findings across data sources (interviews, logbooks, and participant observation notes) and triangulating perspectives... The latter involved validating the ‘bridge’ researcher’s interpretation with ‘inside’ and ‘outside’ researchers, after which the consolidated team findings were cross-validated with research participants via member checking. To further strengthen this process, Artificial Intelligence (AI) was used as a supplementary validation tool..." (p. 6) |
| **Results and Findings** |  |  |
| 16 | Synthesis and Interpretation | The analysis synthesizes participant definitions (p. 7), compares the distinct cultural contexts (p. 9), and contrasts the development trajectories and engagement patterns of the two sites using descriptive text and data visualizations (Graphs 1-4, pp. 13-17). The findings are organized thematically to synthesize and compare key aspects such as partner engagement, development stages, and leadership strategies. |
| 17 | Links to Empirical Data | The findings are substantiated with direct quotes from participants (e.g., Mélanie, Nathalie, Maude, Jean-Baptiste, Sarah, Patricia) throughout the section (e.g., pp. 7-9, 11-12, 14) and are visually represented through data presented in Table 1 (p. 5) and Graphs 1-4 (pp. 13-17). |
| **Discussion** |  |  |
| 18 | Integration with Prior Work & Implications | The Discussion section (pp. 17-20) integrates the study's findings with prior work and discusses their implications. This is achieved by comparing results to other international studies (e.g., “consistent with a comparative study of initiatives in Italy and the UK”), contextualizing them within broader literature (e.g., “strongly supported by reviews of place-based initiatives”), and explaining their relevance to established theories (e.g., “mirrors findings from other complex systems”). |
| 19 | Limitations | "This study has several limitations. Findings may have limited generalizability... Several potential sources of bias should also be noted... while the use of Artificial Intelligence (AI) as a supplementary tool enhanced objectivity, we recognize its inherent limitations." (p. 20) |
| **Other** |  |  |
| 20 | Conflicts of Interest | "Declaration of conflicting interests: No competing interest to declare." (p. 20) |
| 21 | Funding | "Funding: The authors disclosed receipt of the following financial support for the research, authorship, and/or publication of this article: the project is funded by the Fondation J-L Levesque; AB is supported by the Canada Research Chairs program [grant number CRC-2020-00029]." (p. 21) |
